# Genetic parameters for worm resistance in Santa Inês sheep using the Bayesian animal model

**DOI:** 10.5713/ajas.19.0634

**Published:** 2020-01-13

**Authors:** Francelino Neiva Rodrigues, José Lindenberg Rocha Sarmento, Tânia Maria Leal, Adriana Mello de Araújo, Luiz Antonio Silva Figueiredo Filho

**Affiliations:** 1 Federal Institute for Education Science and Technology of Piauí (IFPI), 64.750-000, Paulistana, PI, Brazil; 2 Department of Animal Science, Federal University of Piauí (UFPI), 64.049-550, Teresina, PI, Brazil; 3 Brazilian Agricultural Research Corporation (Embrapa Meio Norte), 64.006-220, Teresina, PI, Brazil; 4 Federal Institute for Education, Science and Technology of Maranhão (IFMA), 65.609-899, Caxias, MA, Brazil

**Keywords:** Famacha, *Haemonchus contortus*, Heritability, Multivariate Analysis

## Abstract

**Objective:**

The objective of this study was to estimate the genetic parameters for worm resistance (WR) and associated characteristics, using the linear-threshold animal model via Bayesian inference in single- and multiple-trait analyses.

**Methods:**

Data were collected from a herd of Santa Inês breed sheep. All information was collected with animals submitted to natural contamination conditions. All data (number of eggs per gram of feces [FEC], Famacha score [FS], body condition score [BCS], and hematocrit [HCT]) were collected on the same day. The animals were weighed individually on the day after collection (after 12-h fasting). The WR trait was defined by the multivariate cluster analysis, using the FEC, HCT, BCS, and FS of material collected from naturally infected sheep of the Santa Inês breed. The variance components and genetic parameters for the WR, FEC, HCT, BCS, and FS traits were estimated using the Bayesian inference under the linear and threshold animal model.

**Results:**

A low magnitude was obtained for repeatability of worm-related traits. The mean values estimated for heritability were of low-to-high (0.05 to 0.88) magnitude. The FEC, HCT, BCS, FS, and body weight traits showed higher heritability (although low magnitude) in the multiple-trait model due to increased information about traits. All WR characters showed a significant genetic correlation, and heritability estimates ranged from low (0.44; single-trait model) to high (0.88; multiple-trait model).

**Conclusion:**

Therefore, we suggest that FS be included as a criterion of ovine genetic selection for endoparasite resistance using the trait defined by multivariate cluster analysis, as it will provide greater genetic gains when compared to any single trait. In addition, its measurement is easy and inexpensive, exhibiting greater heritability and repeatability and a high genetic correlation with the trait of resistance to worms.

## INTRODUCTION

Worm diseases are those that affect sheep and cause productive and economic damage in sheep breeding. The selection of sheep genetically resistant to worms (WR) is a promising alternative in the control of the disease since this trait is inheritable and can be improved in a herd. The WR is the animal ability to avoid infection, reduce parasitic burden, or recover from an infection [1]. This ability is strongly influenced by environmental factors [2] and varies substantially among breeds [3].

The number of eggs per gram of feces (FEC) is the most commonly used information for selection of resistant animals [4]. The FEC is a direct, but a very variable, information about host-parasite load [5]. Due to this variability, other traits may be included to help in the process of identification of resistant animals using multivariate analysis methods, such as the Famacha method [6] and hematocrit (HCT), since verminosis caused by *Haemonchus contortus* is the main cause of anemia in sheep [7]. Body condition assessment can also be applied when weight loss in sheep is caused by parasite load [8]. Thus, ovine selection for these traits requires the knowledge of genetic parameters such as heritability.

In the estimation of genetic parameters for worm-related traits, two situations are commonly found: data with non-normal distribution (e.g., FEC) and categorical variables (e.g., Famacha score [FS]). To estimate the genetic parameters of these traits, Bayesian inference, using the animal threshold model, has been demonstrated to be adequate for this type of variable [9,10].

Bayesian inference does not require normal distribution of data since a probability density function (*a posteriori* distribution) can be calculated on all possible parameter vectors. This can be done from the information included in the analysis, using an *a priori* distribution of the parameters to be analyzed, together with the uncertainty about it [11].

As compared to linear models, the use of threshold models is recommended for traits that assume exhibit discrete distribution because they have a greater ability to detect variability of genetic origin. These models are based on the assumption that the categorical data classes are related to a normal underlying scale [12].

Therefore, the objective of this study was to estimate the variance components and genetic parameters for WR and associated traits, using the linear and threshold animal model, via Bayesian inference, in uni- and multiple-trait analyses.

## MATERIALS AND METHODS

The experimental procedure was approved by the Institutional Animal Care and Use Committee at Federal University of Piauí, Brazil (414/17).

This study was conducted with data collected from the herd of Santa Inês breed sheep (total 516 animals), of both sexes, kept in an experimental farm (“Sol Posto”, Campo Maior, PI, Brazil; a property of Embrapa Meio-Norte) in the period Aug 2012 to Jul 2015. All collections were carried out on animals submitted to the natural conditions of contamination, without any change in the management used in the farm, because conservation of the herd breed was the main objective of the study.

The herd food base was a native pasture where the grass species *Axonopus purpusii* (capim-mimoso) predominates. Mineral supplementation was carried out throughout the year, using a mineral supplement suitable for the species. In the driest season of the year, when food scarcity occurred, the herd received supplementation with voluminous and/or concentrated food.

In the experimental period, the sanitary management comprised worming, vaccination, and treatment of animals that sickened individually for any reason. Vermifuge was administered after the FEC and HCT results.

All data, FEC, FS, body condition score (BCS), and HCT were collected on the same day. On the day after collection, after a 12-h fast, the animals were weighed individually.

The FEC was counted by the method of Gordon and Whitlock [13], modified by Ueno and Gonçalves [5]. Coproculture was performed according to the methodology described by Roberts and O’Sullivan [14].

The anemia degree was evaluated by the Famacha method, observing the color of the ocular mucosa of sheep and assigning a score (1 to 5) according to the Famacha card [6].

In the evaluation of BCS, scores were attributed to the animal based on palpation and visualization of the lumbar region. This was done mimicking pincer movement, with the application of constant pressure around and between the transverse and spinal apophyses. This was also done in the sternum region, and the amount of skin, muscle and fat density was evaluated in both anatomical regions, according to the methodology cited by Ribeiro [15]. The score attributed in the evaluation was based on the perception of fat and muscle deposited in the evaluated regions, based on the one-to-five scale, in which body condition five indicates excessive fat deposition in the animal.

The HCT was determined using the microhematocrit technique. For this purpose, blood samples were collected from the jugular vein, and antisepsis (iodized alcohol) of the site was performed using a needle directly coupled to vacuum tubes containing ethylenediamine tetraacetic acid. Subsequently, the samples were analyzed in the hematological packvet equipment (University Veterinary Hospital of Teresina, Federal University of Piauí, UFPI).

To define the resistance trait, as proposed in this study, the animals were grouped into three classes (resistant, intermediate, and sensitive) and multivariate analysis of the characteristics FEC, HCT, BCS, and FS was performed. The classification was performed with the standardized data, using the K-means algorithm (skmeans package) available in the R language [16]. After the classes were formed, the data were submitted to analysis of variance to determine the differences between the characteristics used in grouping the animals into the three classes attributed to the WR trait. The values for the FEC trait underwent a logarithmic transformation (log_10_[FEC+1]) and were renamed as LFEC.

The database was edited and formatted, using the statistical [17] software. Information on animals with less than three repeated measurements, as well as that of animals without pedigree information, was discarded. The collection months were grouped into two collection seasons: rainy season (Jan–Jul), with greater food supply (ECO_1_) and dry season (Aug–Dec), with smaller food supply (ECO_2_). The birth months were grouped into two periods (EN_1_ and EN_2_), similarly to what was done with the collection seasons. Regarding age, the animals were grouped into four age classes: less than one year (CI_1_); between one and two years (CI_2_); between two and four years (CI_3_); and greater than four years (CI_4_). As for the physiological status, the animals were grouped into six categories (CAT): matrices in gestation (in the first thirds of gestation, CAT_1_); matrices in peripartum (last month of gestation and lactation period, CAT_2_); dry matrices (females after weaning and not pregnant, CAT_3_); pups aged less than six months (CAT_4_); pups aged six months to one year (CAT_5_); and adult males (CAT_6_).

Sex, birth season, age class, year of birth (ANON/YB), and CAT were considered to form the contemporary groups (CG). The effects considered in the CG were tested to be significant (p<0.05). Groups with less than 3 animals were excluded. After consistency analysis, a file containing information about animal, father, mother, CG, collection year and station, FEC, HCT, BCS, FS, and body weight (BW) was edited. After formatting, the file contained 2,753 observations and 427 animals in the numerator matrix for the Wright’s kinship coefficients.

The variance components and genetic parameters for the WR, FEC, HCT, BCS, and FS traits were estimated using Bayesian inference under the linear and threshold animal model, using the THRGIBBSF190 application [18] in uni- and multiple-trait analyses. This last analysis was performed combining four traits: LFEC and HCT were used as anchor characteristics, and resistance, FS, and BCS were varied. For every analysis, a Gibbs chain of 4,000,000 samples (with burn-in of the first 2,000,000 samples and sampling interval for every 200 samples) was generated, resulting in an *a-posteriori* distribution with 10,000 samples, from which inferences were made.

The values for burn-in and sampling interval were defined based on preliminary analyses, in which convergence and distribution of samples were evaluated using the POSTGIBBS1F90 program [18]. In this program, the Geweke’s [19] diagnostic test was used, taking the Z test (for equality of means of the logarithm of the conditional data distribution) as the basis.

The animal model can be represented in matrix notation as follows: *y* = *Xβ*+*Zα*+*Wγ*+*ɛ* (1); where: *y* is the observation vector of the study traits; *X* is the *n*×*f* incidence matrix (n is the total number of observations, and *f* is the number of systematic effect classes), relating observations to systemic effects; *β* is the vector for systemic effects of contemporary groups (formed by sex, CI, EN, year of data collection, and CAT); *Z* is the *n*×*N* incidence matrix, relating direct additive genetic effects, where *n* is the total number of observations and *N* is the number of individuals in the numerator matrix for Wright’s kinship coefficients (427); *α* is the vector of direct additive effects in each animal (genetic value); *W* is the *n*×*N* matrix of mean permanent environment effects; *γ* is the mean permanent environment effect vector; and *ɛ* is the residual random error vector associated with each observation.

In the Bayesian analysis, the systemic and random effects included in the model are considered random variables. Under the Bayesian approach, the information (*y*) and data (*β*, *α*, *γ*, *e*, $\sigma_{e}^{2}$), which assume a multivariate normal distribution, accept the assumptions as follows:

$$\begin{array}{l} y|\beta ,\alpha ,\gamma ,\sigma_{e}^{2}\sim N(X\beta +Z\alpha +W\gamma +ɛ)\\ \alpha |A,G\sim N(0,A⊗G_{0})\\ \gamma |I,P\sim N(0,I⊗P_{0})\\ e|I,R\sim N(0,II⊗R_{0}) \end{array}$$

where *G*_0_, *P*_0_, and *R*_0_ are the direct additive genetic (co)variance matrices and permanent and residual environments, respectively; *A* is the numerator matrix of Wright’s kinship coefficients and *I* is the identity matrix of order equal to the number of animals with observations. For *β*, non-informative *a priori* and p(*β*) ∝ constant were assumed.

Convergence was monitored using the criteria of Geweke [19] and error of the Monte Carlo chain. The variance of samples taken from each component was divided by the number of samples to obtain the Monte Carlo error (MCE). Thus, the square root of this value refers to the approximation of the standard deviation of the error associated with the Gibbs chain size [20].

For the threshold model, FS, BCS, and WR traits were assumed. Normal distribution was also assumed for the underlying scale, which is represented as: $U|\theta \sim N(W\theta ,I\sigma_{e}^{2})$, where: *U* is the vector of the base scale of origin r; *θ* (*b*, *a*) is the vector of the location parameters of order s with a (direct additive random effect); *W* is the incidence matrix of order *r*×*s*; and $\sigma_{e}^{2}$ is the residual variance.

The conditional probability that *y_i_* falls into category *j* (1, 2, 3, 4, 5 [FS]; 1, 2, 3, 4, 5 [BCS]; and resistant, partially resistant, and sensitive [resistance]), given the vectors *β*, *a*, and *t* (*t* = *t*_min_, *t*_1_, ..., *t*_j-1_, *t_máx_*), is represented as:

$$\begin{array}{l} \text{Pr}(y_{i}=j|\beta ,a,t)=\text{Pr}\left(t_{j-1}<U_{i}<t_{j}|\beta ,a,t\right)\\ =\Phi \left(t_{j}-{X^{\prime }}_{i}\beta -{z^{\prime }}_{i}a-{z^{\prime }}_{i}c\right)-\Phi \left(t_{j-1}-{X^{\prime }}_{i}\beta -{z^{\prime }}_{i}a-{z^{\prime }}_{i}c\right)\\ =p(y_{i}|\beta ,a,t). \end{array}$$

Since residual variance is not estimable in threshold models, the value 1 was assigned to it in the parameterization process to obtain identifiability in the likelihood function [12]. Such an assumption is a standard procedure in categorical data analyses. Flat (non-informative *a priori* distribution) was defined for all initial variances.

## RESULTS AND DISCUSSION

The coproculture analyses showed that the genus *Haemonchus* was predominant in all collections (mean, 83%; variation, 72% to 95%). *Trichostrongylus* was the second most prevalent genus (12%) followed by *Oesophagostomum* (5%), and other trichostrongylids were not found. Prevalence of the genus *Haemonchus* was confirmatory to the study because anemia indicator-parameters were evaluated, and this parasite is the main causative agent of anemia in sheep [7]. In addition, it is the main parasite of small ruminants in tropical and subtropical regions [2]. It is worth highlighting the life cycle characteristics of *Haemonchus* sp., which can survive to high temperatures with high fecundity and may resist to antiparasitic drugs. These characters indicate that this species has a large potential for incidence in the edaphoclimatic conditions of the region where this study was carried out.

The mean values for all these variables are presented in Table 1 and are considered normal for the study traits. In the FEC and HCT variables, the mean value was within the normal values for the traits but with a large (middle-to-high) dispersion in the variation coefficients. Given the existence of the phenotypic variation, this is a necessary condition to include these variables in a breeding program.

The mode value for the BCS trait (Table 1) indicates good food availability for the animals evaluated and, consequently, a good muscle and fat composition in the lumbar region.

The mode values were normal and even higher than the mean values for each variable, indicating that variation occurred in the results as a function of a small number of animals (Table 1). According to Pereira [21], the qualitative characteristics of categorical characters (e.g., FS, BCS, and WR) are better represented by the central tendency statistics (median and mode).

In the resistance level classification, which was obtained using clustering analysis, two groups showed significant differences. Among all parameters analyzed, the animals that presented the best results (i.e., lower FEC and higher HCT values, higher BCS and lower FS) were classified as resistant. The other animals, which presented opposite results, were classified as sensitive. The third group presented varying results: FEC values equal to those of the resistant group, BCS values equal to those of the sensitive animals, and HCT and FS differing from both groups. Thus, as these animals presented characteristics common to both groups, they were classified as partially resistant (Table 2).

In the convergence analysis of the chain using the Bayesian method, convergence was achieved for all characteristics studied. The sample chain size used was also sufficient to obtain the *a-posteriori* estimates of marginal distributions since the Geweke’s criterion [19]. In addition, the MCE presented low values, indicating convergence. According to Van Tassel and Van Vleck [20], there is indication that convergence was achieved when the error value and average estimate of the *a posteriori* distribution of the heritability coefficient are summed and change in the value magnitude of this estimate (up to the second decimal place) is not observed (Table 3).

A change was observed in the values estimated for the (co)variance and heritability components of the study traits (Table 3) due to the contribution of the multiple-trait analyses to both the increase in the amount of information taken into account and influence of correlations between the study traits. This indicates a recovery in a portion of the additive genetic variance that was incorporated into the residual variance in the uni-traits analyses (Table 3), thus confirming what was observed by Sarmento et al [22]. Therefore, the multiple-trait model may be the best choice, since it allows removing the bias caused by sequential selection [22] and obtaining best estimates of genetic components from the phenotype [23].

The repeatability coefficients observed in the genetic parameter estimates of the study traits using multiple-trait analysis (Table 3) varied in the range of low-to-high (0.12 to 0.59) magnitude. Values of low-to-moderate magnitude have also been reported by Prince et al [24] for the FEC trait. These results indicate that the probability of a single measure of the animal phenotype to represent its genotype is low since these traits undergo environmental temporary changes. The lowest repeatability (0.12) was observed in the FEC variable, although this trait undergoes great changes in the life of animals [5]. This variance is mainly related to the environment, as found in this study, in which 82.4% of the phenotypic variance is of environmental origin.

By using the values estimated by multiple-trait analysis, heritability values with low-to-high (0.10 to 0.88) magnitude were observed. The FEC, HCT, BCS, FS, and BW presented low heritability values (Table 3), whereas the resistance trait presented a high (0.85) heritability value. This result indicates that in this herd the genetic gain for selection of the WR trait obtained in the group (FEC, HCT, BCS, and FS) is greater than that obtained with the individual variables.

Different heritability values were obtained due to various (either genetic or environmental) factors. Greater control over the environmental factors can reduce the environmental variance, increasing the percentage of phenotypic variance explained by the additive genetic variance [25].

The low heritability estimated for HCT, as obtained in this study, was also reported by Gauly et al [3]. These low-magnitude values for the heritability estimates can be attributed to the variation among breeds [3]. The heritability values for the FS found in this study are similar to those (0.06 to 0.24) estimated by Ryley and Van Wyk [6].

Regarding the WR trait, the value for heritability obtained in the multiple-trait analysis (0.88) was higher than that obtained in the uni-trait analysis (0.44) (Table 3). The variables FEC, HCT, BCS, and FS also showed a slight increase in the heritability values with the use of the multiple-trait method (Table 3). However, their magnitude remained low, indicating that the models for these variables have close efficiencies. The increase in the values estimated for heritability of the FEC and HCT variables, as observed in multiple-trait analysis, is due to the decrease in values for permanent-effect variance and increase in those for additive genetic variance. Thus, the model allowed to recover part of the additive genetic effect that was considered as a non-additive genetic effect.

On the other hand, an increase in values for permanent-effect variance occurred in the BCS and FS variables. This occurred because all information about the study trait is used in the multiple-trait model, but the BCS and FS are strongly influenced by the parasitic load [8,26]. This result indicates that part of the variation in animal response to the parasitic load, as expressed by BCS and FS, has a genetic but non-additive nature.

The genetic correlations between the study traits are shown in Table 4. The WR trait showed a high and favorable correlation with the FEC, FS, HCT, and BCS variables, indicating the possible use of any of the traits via correlated response to improve WR in Santa Inês sheep.

The FEC values showed low genetic correlations with the HCT, FS, BCS, and BW variables, in agreement with Lôbo et al [27] who evaluated Santa Inês sheep in the Sergipe State (Brazil) and found genetic correlation values close to zero. Low correlations between these variables may be related to the fact that they are responses to the FEC values, but the observed changes are not usually immediate but late, making association difficult. In addition, the HCT, FS, and BCS traits are highly influenced by other factors, which are different from the parasitic load. These are mainly linked to food and sanitary management, which may help explain these low genetic correlations.

The genetic correlations between BW and the other variables were unfavorable. These results indicate that improvement for WR in this herd will cause few changes in the BW of adult animals. The inverse situation also occurs, i.e., genetic progress for WR will not be observed if selection for BW is performed. This may explain the frequent occurrence of sanitary problems with verminosis in herds of Santa Inês sheep, since for a long time the objective of cattle breeders was to select animals by their ponderal performance and body size, and these traits contributed very little to the genetic improvement of resistance to gastrointestinal parasites.

On the other hand, FS showed favorable correlation with all variables, and the correlation with the WR trait was the largest of them. Thus, as FS is highly correlated with the WR and HCT traits and a mean correlation with BCS, it becomes an interesting variable to be included in genetic improvement programs with a view to select worm-resistant sheep. In addition, this trait is still of easy and inexpensive measurement.

The high correlation (−0.87) between FS and HCT indicates that genes related to mucosal color and globular volume are strongly associated, and the Famacha method represents well the clinical anemia in sheep. The FS and BCS presented a mean genetic correlation (−0.54). Values for genetic correlation between these variables were not found in the literature, and some authors have reported the existence of a negative (low-to-middle) sample correlation between these variables [28–30]. This result indicates that the best body condition (higher BCS), exhibited by animals with the most colored mucosa (lower FS), is not due only to environmental causes (e.g., nutrition), but also to an additive genetic effect.

The moderate genetic correlation (0.54) between BCS and WR character indicates that genetic gain for WR will occur in the sheep herd when BCS, which is commonly included as a criterion for animal selection for carcass quality, is used in selection programs.

## IMPLICATIONS

The worm resistance trait was determined by multivariate clustering analysis and showed a high estimated heritability, which can be improved by selection. The Famacha score is recommended for inclusion as a criterion for genetic selection of sheep in order to improve their resistance to endoparasites, since its measurement is easy and inexpensive, presents greater heritability and repeatability, and a high genetic correlation with the worm resistance trait.

## Figures and Tables

**Table 1:** Descriptive statistics of the traits studied in Santa Inês sheep

Traits	n	Mean	Mode	CV	Minimum	Maximum	Range
FEC	2,753	1,217.4	0.0	189.9	0.0	24,500.0	24,500.0
HCT	2,753	29.7	30.0	15.3	10.0	43.0	33.0
BCS	2,753	2.4	2.0	39.2	1.0	5.0	4.0
FS	2,753	2.4	3.0	38.1	1.0	5.0	4.0
BW	2,753	43.6	50.0	24.4	13.0	72.5	72.5

CV, coefficient of variation; FEC, number of eggs per gram of feces; HCT, hematocrit; BCS, body condition score; FS, Famacha score; BW, body weight.

**Table 2:** Classification of animals according to criteria for resistance to worms

Classification	FEC (LFEC)	HCT (%)	BCS	FS	BW (kg)
Resistant	873.2 (2.1^B^)	31.3^A^	2.7^A^	2.0^C^	39.4^C^
Partially resistant	1,059.5 (2.2^B^)	28.3^C^	2.2^B^	2.7^A^	45.9^A^
Sensitive	2,906.6 (2.8^A^)	28.8^B^	2.1^B^	2.4^B^	44.2^B^

FEC, number of eggs per gram of feces; LFEC, logarithm-transformed FEC; HCT, hematocrit; BCS, body condition score; FS, Famacha score; BW, body weight.

A–C In the columns, mean values followed by the same letter do not differ by the Student’s t (5% probability; for FEC, HCT, and BW) and Kruskal-Wallis (for BCS and FS) tests.

**Table 3:** Estimates of variance components and genetic parameters of traits linked to resistance to worms in uni- and multiple-trait analyses

Traits	$\sigma_{a}^{2}$	$\sigma_{pe}^{2}$	$\sigma_{e}^{2}$	$\sigma_{p}^{2}$	r	*h*^2^(SD)	Geweke	MCE
Uni-trait								
WR	0.37	-	0.47	0.83	-	0.44 (0.23)	0.00	0.0043
FEC	0.18	0.15	3.44	3.76	0.09	0.05 (0.03)	−0.01	0.0171
HCT	1.49	1.87	15.74	19.10	0.18	0.08 (0.05)	−0.01	0.0003
BCS	0.06	0.02	0.41	0.50	0.17	0.12 (0.05)	0.00	0.0003
FS	0.05	0.04	0.26	0.36	0.27	0.15 (0.07)	−0.05	0.0004
BW	8.72	31.02	33.11	72.85	0.55	0.12 (0.09)	0.04	0.1100
Multiple-trait								
WR	0.76	-	0.38	1.14	-	0.88 (0.03)	0.00	0.0043
FEC	0.07	0.02	0.67	0.75	0.12	0.10 (0.01)	−0.01	0.0171
HCT	2.56	0.96	15.85	19.37	0.18	0.13 (0.03)	−0.01	0.0003
BCS	0.09	0.03	0.42	0.34	0.23	0.17 (0.03)	0.00	0.0003
FS	0.08	0.05	0.23	0.34	0.33	0.23 (0.04)	−0.05	0.0004
BW	19.03	28.00	32.62	79.65	0.59	0.22 (0.06)	0.04	0.1100

$\sigma_{a}^{2}$, additive genetics; $\sigma_{pe}^{2}$ permanent environment; $\sigma_{e}^{2}$, residual variances; $\sigma_{p}^{2}$, phenotypic variances; r, repeatability; *h*^2^, heritability, SD, standard deviation; MCE, Monte Carlo error; WR, worm resistance; FEC, number of eggs per gram of feces; HCT, hematocrit; BCS, body condition score; FS, Famacha score; BW, body weight.

**Table 4:** Mean estimates (standard deviation) for genetic correlation between traits linked to resistance to worms in Santa Inês sheep

Traits	FS	HCT	WR	BW	BCS
FEC	0.17 (0.15)	−0.18 (0.23)	−0.66 (0.06)	0.05 (0.35)	−0.17 (0.14)
FS	-	−0.87 (0.27)	−0.67 (0.07)	0.51 (0.29)	−0.54 (0.20)
HCT	-	-	0.62 (0.12)	−0.09 (0.33)	0.71 (0.19)
WR	-	-	-	−0.42 (0.15)	0.54 (0.08)

FS, Famacha score; HCT, hematocrit; WR, worm resistance; BW, body weight; BCS, body condition score; FEC, number of eggs per gram of feces.
